# Simple Remedies

**Published:** 1883-03

**Authors:** 


### SIMPLE REMEDIES.

CONTINUED FROM THE JANUARY NUMBER.

NO one truly realizes the extent of suffering endured every hour of every day, and of the lives miserably sacrificed because of thoughtlessness or through ignorance of the value of such simple remedies as are almost always within the reach of every person. In our January number we mentioned some of the uses of salt, an article that is always at hand and which, in case of poisoning, can be at once given as an emetic, a tablesponful in a tumbler of cold, or, better still, tepid water, and which will promptly relieve the stomach of its contents. But suppose, as might sometimes happen, even so simple an article as salt was not quickly to be had? Then tepid water will probably answer every purpose; a pint or even a quart should be drank, and the palate should be tickled with the finger, when vomiting will quickly follow. Using the finger in this way will often induce vomiting without resorting to other means.

When a person has swallowed strong acid, except sulphuric acid, by mistake, as occasionally happens, chalk, magnesia or soda should be put in water and drank as soon as possible to neutralize the acid. Water with sulphuric acid generates heat and makes the matter worse—in that case use magnesia with oil or grease, and afterward in all these cases an abundance of water should be taken into the stomach to induce vomiting. Warm water is used to-day more extensively in hospitals than any other remedy. Cotton dipped in warm water and applied to old sores and ulcers, will heal them sooner than any other remedy and with far greater certainty. Felons and other painful abscesses do not long resist the incessant application of this simple remedy. When threatened with a felon, the finger should be held in a dish of water, as warm as it can be comfortably endured, and kept there till the pain and throbbing cease. In this way it can be cut short. We have known a sprained ankle which had been painful for weeks, cured in an hour by letting a stream of water—just hot enough not to burn—run on it for that length of time. No remedy affords such prompt relief in croup as a piece of flannel wrung out in hot water, and wrapped around the little sufferer's neck. Two or three thicknesses of flannel wrung out in hot water and spread over the chest is an excellent treatment in congestion of the lungs, particularly of children, and the same treatment is useful in colic. This is also an excellent remedy for fixed pains in the joints or muscles of the body, and for all local inflammations except piles, where cold water seems usually to be more effective. For general bathing, the temperature of the water should not be lower than that of the air of the bath room.

For weak eyes, the careful use of warm water affords great relief. If the eyelids are closed, and a few drops of bay rum or brandy or whiskey are applied to the eyelids with the finger, it seems to greatly strengthen eyes that are weak and watery. It is of advantage to let a little of the vapor of the liquor reach the eye. It stimulates it and makes it feel comfortable after the slight, momentary smarting stops. If alcohol is used, it should be diluted with water one-half. Hartshorn is a remedy most valuable as an application for bites and stings, and a glass stoppered bottle of it should be kept in every house—but beyond the reach of small children.

Now the best of *remedies* in many cases of accidents are a cool head and a knowledge of what should be done.

There is no immediate danger from severing a superficial vein of considerable size ; but not so with arteries. When an artery is cut the red blood spirts out in a jet at each pulsation. The first thing to be done is to check the bleeding until the doctor comes. This can always be done by pressing the thumb on the artery above the wound—that is, between the wound and the heart—or else, if the wound is small, pressing the thumb firmly on the wound ; this stops the bleeding, and stops the flow of blood through that artery below the point of pressure. If the wound is where it can be bandaged, use a compress, which may be a small stone, piece of wood or other hard substance ; put this on the wound and tie a bandage or pocket handkerchief around to produce pressure and keep the stone in position so that the bleeding can't go on.

If a person could forget that the wounded was a human being and imagine that he was stopping a leak in a cask, he would do it coolly and effectually. It is the responsibility that unnerves us. A cool head is best for heroic deeds.

If a person receives a fracture of the skull, life often depends on at once raising up the splintered edges of the wound, so as to reliev, pressure on the brain. Any one might do this with the blade of a pocket knife, or even a nail, and thus, perhaps, save a valuable life, if he knew that it was necessary and had presence of mind.

Thus all can appreciate the value of such knowledge as may be utilized at any time in the relief of suffering and the saving of life, since various accidents are constantly occurring on every side of us. Without such knowledge no one is safe, for one hour, and he can give little aid to others in case of need.

If a person is thrown from a wagon in the street and severely injured, hundreds, perhaps, will gather around him ; and although he may be bleeding to death from a wound that could be instantly compressed with the thumb and the bleeding stopped, the chances are that not not one person will come to his relief, because not one will know what should be done. In Dr. Hall's Health at Home a case is mentioned where a child of eight years accidently tapped the main artery of the leg with the blade of a pocket knife. One member of the family hastened to call a physician, while the others wept bitterly as they watched the life blood flowing away, and not knowing what to do, they did nothing to check the flow of blood. When the doctor arrived the child was dead.

Persons have slowly bled to death after having a tooth extracted, because the ordinary methods of checking the flow have proved unavailing. We have never failed to stop the bleeding in such cases by shaving down a cork so that one end will enter the cavity, and then having the patient close his mouth so as to press the cork down firmly.

